# Synthesis of Highly Crystalline Multilayered Boron Niride Microflakes

**DOI:** 10.1038/srep21403

**Published:** 2016-02-19

**Authors:** Pervaiz Ahmad, Mayeen Uddin Khandaker, Yusoff Mohd Amin, Nawshad Muhammad

**Affiliations:** 1 Department of Physics, Faculty of Science University of Malaya 50603 Kuala Lumpur Malaysia; 2 Interdisciplinary Research Centre in Biomedical Materials (IRCBM) COMSATS Institute of Information Technology, 54000 Lahore, Pakistan

## Abstract

Boron niride microflakes of 2–5 μm in diameter and greater than 40 μm in length with multilayer structure and highly crystalline nature are synthesized in two states of catalysts and dual role of nitrogen at 1100 °C. Most of the microflakes are flat, smooth and vertically aligned with a wall-like view from the top. Transmission electron microscopy shows overlapped layers of microflakes with an interlayer spacing of 0.34 nm. The h-BN components of the synthesized microflakes are verified from B 1s and N1 s peaks at 190. 7 and 397.9 eV. Raman shift at 1370 (cm^−1^) and sharp peaks in the XRD pattern further confirm the h-BN phase and crystalline nature of the synthesized microflakes. Microflakes of h-BN with the above characteristics are highly desirable for the development of a solid state neutron detector with higher detection efficiency.

Hexagonal boron nitride (h-BN) is a very important material with remarkable physical properties and chemical stability. It consists of sp^2^-bonded boron and nitrogen in two dimensional (2D) layers. Boron and nitrogen in the layers are bounded together by strong covalent bond whereas, weak van der walls forces are responsible for holding different layers in h-BN like graphite^1^. Unlike graphite, h-BN is a wide band gap semiconductor^2^ with a direct band gap of 5. 97 eV^3^. The layered structure of h-BN has high temperature stability, thermal conductivity, hardness, mechanical strength, corrosion resistance and low dielectric constant. All these properties have made h-BN a suitable candidate for a wide range of potential applications as structural and electronic material. As a large band gap semiconductor or insulator, h-BN can be used in the electronic equipment as a charge leakage barrier layer. High quality flakes of h-BN have applications in far-ultraviolet light emitting diodes^4^. Solid state neutron detectors with an improved detection efficiency is one of the most promising potential application of h-BN. Such a detector is highly sought for a wide range of applications in fissile materials sensing, neutron therapy, medical imaging, materials science and oil exploration etc.^5^. Nowadays, ^3^He detectors are used for all these purposes. However, along with other difficulties, there is a significant shortage of ^3^He gas^6^. This seriously felt the need for the development of a solid state neutron detector. Such a detector is sought to be made of h-BN film in few micron thickness and highly crystalline nature.

The excellent properties and potential applications of layered structure of h-BN have got the attentions of many researchers in the same field. It was found that few layered h-BN can be exfoliated from bulk crystals of h-BN via some mechanical^7^ or chemical procedure^8^. However the flakes size obtained via such a techniques hinder its use for further applications^4^. Chemical vapor deposition (CVD) is nowadays, the most commonly used technique for obtaining thin films of h-BN. Initially in this technique BF_3_/NH_3_^9^, BCL_3_/NH_3_^10^, or B_2_H_6_/NH_3_^11^ were used as precursors. In these precursors, the molar ratio of boron and nitrogen from their respective sources were not only unable to maintain their stoichiometric ratio in h-BN layers but also affected the deposition rate. High quality h-BN thin film has also been synthesized^4^ from borazine (B_3_N_3_H_6_) as a precursor via atmospheric pressure chemical vapor deposition (APCVD). However, the B_3_N_3_H_6_ used as precursor is not only toxic but also resulted in few layer thin film of h-BN in the range of 5–50 nm^4^.

In the previous works^4^,^9^,^10^,^11^, the toxicity of precursors, complicated and lengthy experimental procedures and lower quality of the final products were some of the main problems which have limited the overall progress in the synthesis of pure structure of h-BN. Some of the researchers^12^,^13^,^14^ have also claimed a simple technique for the synthesis of nanostructures of h-BN. However, in their developed techniques, NH_3_ has been used as a nitrogen source with other precursors^12^,^13^,^14^. During the growth, NH_3_ is flown in to the system with a flow rate of 200 sccm at 1200 °C^13^. At this temperature, NH_3_ decomposes, provides nitrogen for BNNTs synthesis and generates H_2_O(g) and H_2_(g)^3^,^15^. Here, it should be noted that all of the NH_3_ is not decomposed during the growth. The undecomposed NH_3_ can produce serious health problem when it emits or leaks from the system in to the atmosphere. Thus extra safety precautions are needed to avoid such a health risk factors. Furthermore, during the experiment, the as-produced H_2_(g) can cause a sudden increase in the internal temperature of the system which can not only results in the destruction of the synthesized samples but also of experimental set up. Moreover, it has been mentioned that the system was evacuated to a certain level during the experiment, but, it is not clearly mentioned whether the evacuation was maintained till the end or only for a certain moments in the beginning. In both the cases it may result in the failure of the experimental process. Thus the previous work shows lack of critical experimental information due to which is very difficult to be followed by the other researchers. Also the same technique has been claimed for the synthesis of nanotubes^13^ and nanosheets^14^. Thus, it creates further confusions to the fact that exactly which product can be synthesized with their mentioned technique.

Therefore, in the present study, we develop a straightforward technique for the synthesis of h-BN microflakes (BNMFs) with well-known experimental parameters and simple logics. Maximum efforts have been made to avoid the use of toxic precursors, and make the experimental process simple and safe. In the experimental process, B, MgO and γ-Fe_2_O_3_ are used as precursors and N_2_ as a reaction atmosphere and nitrogen source. At lower temperature, N_2_ provides an inert atmosphere whereas at higher temperature (1100 °C), it can easily dissociates in the presence of the as-produced Mg and Fe catalysts, and provides nitrogen for the formation of microflakes. The use of the nitrogen in the present work not only makes the overall process simple and relatively shorter time consuming but also safe and better in comparison to any other technique.

## Experimental details

Microflakes of h-BN are synthesized in the presence of nitrogen as a reaction atmosphere. Micron size powder of B, MgO and γ-Fe_2_O_3_ are used as precursors. Dual zone quartz tube furnace with a smaller one end closed inner quartz tube is used as an experimental set up^16^. In the experimental process, the precursors (of total weight 200 mg) are homogeneously mixed (in 2:1:1 ratio) in alumina boat. The precursors in the boat is partially covered with a few Silicon (Si) substrates and placed inside one end closed quartz tube. The tube is then seal closed in quartz tube chamber of the furnace. The experimental set up is then flushed with Argon gas to remove the dust particles and oxygen from the system. Afterward, nitrogen gas flow is introduced into the system with a flow rate of 200 sccm and standard atmospheric pressure of 1 atm (101325 Pa). The system is then heated up to 1100 °C with a heating rate of 10 °C/ min. 1100 °C is fixed as a final temperature. At this temperature, the system is maintained for 1-h in the presence of nitrogen gas flow.

After 1-h, nitrogen gas flow is stopped and the system is brought to room temperature in the presence of Argon gas flow. At room temperature, Argon gas flow is stopped and the sample is collected from the system for morphological, structural and compositional analysis. The analysis of the characterized results are shown and discussed in the next section.

## Results and Discussion

**Figure 1:**
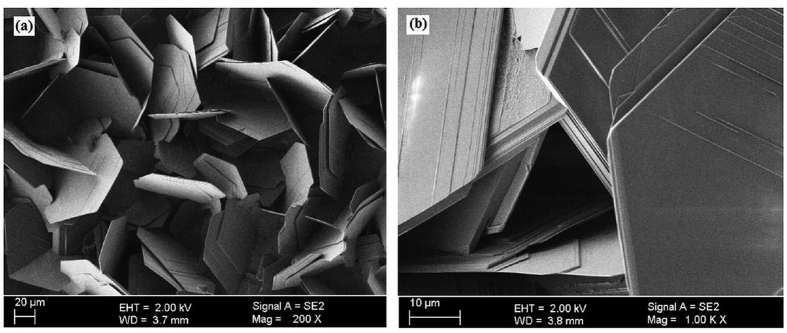
FESEM (**a**) low and (**b**) high magnification micrographs of the synthesized BNMFs in the presence of nitrogen as a reaction atmosphere.

The morphology of the as-synthesized BNMFs is analyzed in low and high magnification with the help of Field mission scanning electron microscope (FE-SEM). The results thus obtained via FE-SEM characterization are shown in Fig. 1. Randomly aligned BNMFs can be seen in the low magnification FE-SEM micrograph shown in Fig. 1(a). Most of the BNMFs are flat and smooth. Some of the BNMFs are vertically aligned and provids a wall-like view from the top. These wall-like BNMFs seem to provide support to the nearby BNMFs and hold them in a particular position. The as-synthesized BNMFs have thickness or diameter in the range of 2–5 μm and length of greater than 40 μm. The results indicate that a layer by layer thick film of h-BN is the initial form of the as-synthesized BNMFs. At higher temperature when the precursors blockage stopped the formation of further growth species, the already form h-BN thick film cracked and acquire the current shape of BNMFs. High magnification micrograph of the as-synthesized BNMFs is shown in Fig. 1(b). In this micrograph some parallel lines can be seen on the edge of each BNMF that proceed towards their centres. However, these cannot be reflected as roughness of the BNMF’s surface. Rather, they can rightly be considered as predecessor for the division of larger size BNMF into further layers of smaller BNMFs. The idea of these division and formation of thinner BNMFs can clearly be seen and observed from the central part of Fig. 1(b). At the central part of the micrograph, each BNMF is found to have a cutting edge. The views of these cutting edges further rectified the existance of parallel lines on the outer surfaces of the BNMFs as the predecessor for the formation of smaller size BNMF.

**Figure 2:**
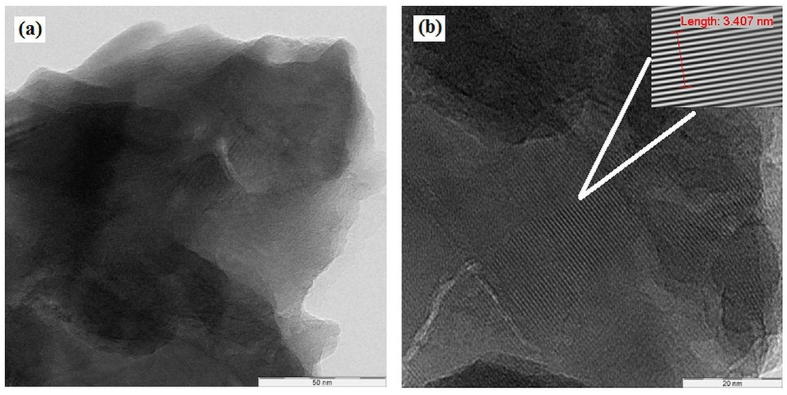
(**a**) TEM shows overlapped layers of BNMF. (**b**) High resolution TEM shows different BNMF’s layers with the lattice fringes on their outer surfaces. The inset shows that the lattice fringes have an interlayer spacing of 0.34 nm.

Transmission electron microscopy (TEM) is used to further study the morphology of the synthesized BNMFs. Low and high resolution TEM micrographs obtained via TEM characterization is shown in Fig. 2. The layers-like structure reported via FESEM characterization of the BNMFs can be confirmed from the low resolution TEM micrograph shown in Fig. 2(a). The dark and light black colors show different overlapped layers of BNMFs. The change in the colors indicates the nature and length of different BNMFs layers. This, in other words, means that all of these layers might have grown in different time intervals. To further analyze the structure, the same BNMF is also analyzed in high resolution and shown in Fig. 2(b). Here, different BNMFs layers can clearly be seen along with the lattice fringes on their outer surfaces. These fringes have a separation of 0.34 nm from one another. These lattice fringes are magnified from a particular location indicated via a white ray and shown as inset on the upper right hand corner of Fig. 2(b). This seperation or inter layers spacing is the characteristics of d_(002)_ plane of h-BN and its highly crystalline nature^2^,^3^.

**Figure 3:**
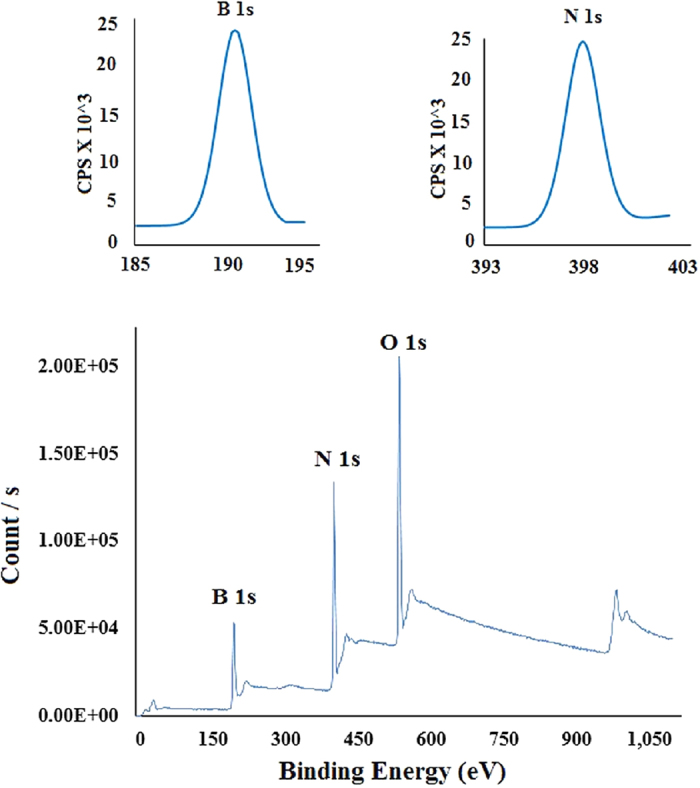
XPS survey of the as-synthesized BNMFs. The inset (upper left and right) shows B 1s and N1s XPS spectra centred at 190. 7 and 397.9 eV.

Boron and nitrogen in the synthesized BNMFs sample are stuided by X-ray photon spectroscopy (XPS). The finger prints of the elements found in the synthesized sample are shown in the XPS survey in Fig. 3. B 1s and N1 s peaks are reported at 190.7 and 397.9 eV. Both of these peaks indicated the presence of h-BN according to the available literature^17^,^18^. To investigate the possibility of existence of other materials (Fe, Mg, MgO or FeO) in the synthesized BNMFs sample, high resolution N 1s and B 1s XPS spectra of the as-synthesized BNMFs are also obtained. These are shown as inset in the upper left and right hand corner of Fig. 3. The non-decomposed and Gaussian nature of both the spectra^19^, deny the existence of other materials as impurities. The XPS survey also reported a high intensity peak at 531 eV. This high intensity peak may be due to the as-used Si substrate^19^.

**Figure 4:**
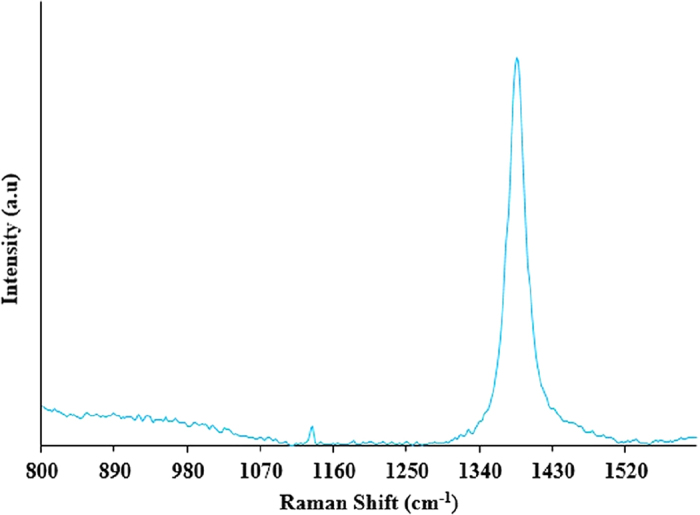
Raman spectrum of the as-synthesized BNMFs shows a Raman shift at 1370 (cm^−1^) that corresponds to E_2g_ mode of vibration in h-BN.

The elemental compositions of the as-synthesized BNMFs reported via XPS are further confirmed by Raman spectroscopy. Raman spectrum of the BNMFs is obtained in the spectral range of 800–1600 (cm^−1^) by using a laser excitation of 514 nm. Fig. 4 shows a Raman shift at 1370 (cm^−1^) that is similar to E_2g_ mode of vibration in h-BN^13^. A smaller intensity Raman shift can also be found in the Raman spectrum shown in Fig. 4 at 1122 (cm^−1^). This smaller intensity shift refers to the presence of boric acid. This might have formed by the interaction of laser with moisture and oxygen in the air and some of the boron-based species found in the sample^20^.

**Figure 5:**
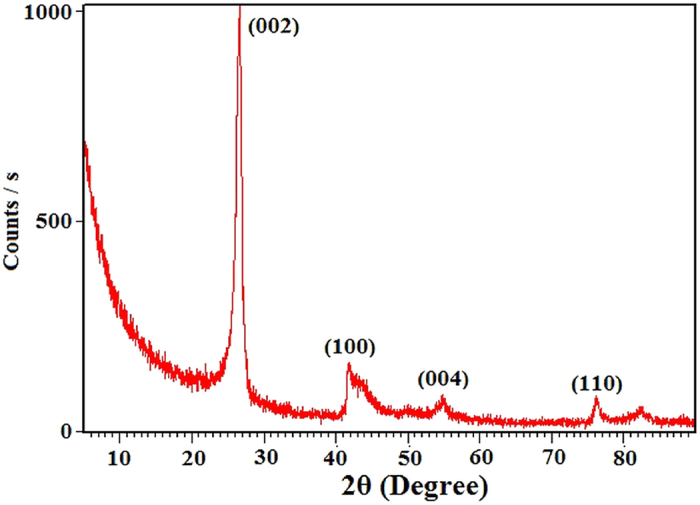
XRD pattern of the as-synthesized BNMFs.

X-ray diffraction (XRD) pattern of the as-synthesized BNMFs sample is taken to further confirm its h-BN phase and crystalline nature. The as-obtained pattern is shown in Fig. 5. The pattern shows a high intensity peak at 26.6° for (002) planes and lower intensity peaks at 41.7°, 54.9° and 76.2° for (100), (004) and (110) planes respectively. The peaks locations in the XRD pattern corresponds to h-BN phase whereas their sharpness represents the highly crystalline nature of the synthesized BNMFs^12^.

**Figure 6:**
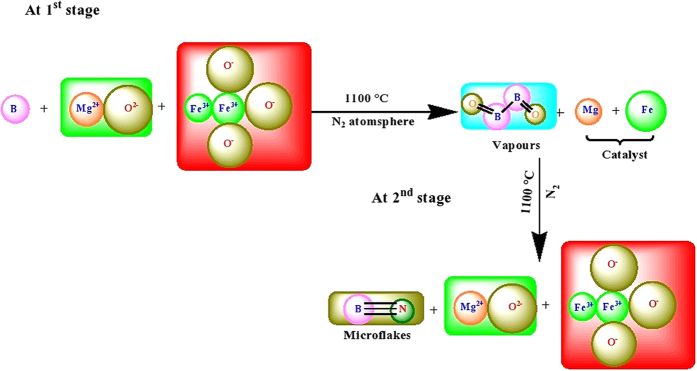
Illustration of the chemical reactions occurred during the synthesis of BNMFs.

Formation of B_2_O_2_ is one of the basic product forms when MgO and γ-Fe_2_O_3_ are used as precursors with B. The formation of B_2_O_2_ is the initial step in the synthesis of h-BN products. For this purpose, MgO and γ-Fe_2_O_3_ work as catalysts with B. Both MgO and γ-Fe_2_O_3_ are effective producers of B_2_O_2_ and catalysts^12^. Thus MgO and γ-Fe_2_O_3_ not only work as catalysts with B to produce B_2_O_2_ but also produces Mg and Fe particles^3^. These catalysts have effective roles in the dissociation of molecular nitrogen and formation of BNMFs. The overall chemical reactions thus happened can be described in the following two stages, as shown in Fig. 6. In the 1^st^ stage the precursors are heated up to 1100 °C in the presence of N_2_ atmosphere. During this stage, N_2_ works as an inert atmosphere inside the reaction chamber. During this stage the precursors synthesize B_2_O_2_ vapors and the metalic catalysts. The as-formed catalysts are adsorbed at the substrate surface whereas B_2_O_2_ remains suspended in the vapor form. Once the metalic catalysts are formed it changes the role of N_2_ in the further experiment. The as-formed catalysts dissociates the molecular nitrogen^21^ and includes it as a precursors in the next stage. In the 2^nd^ stage, the dissociated nitrogen reacts with B from B_2_O_2_ and forms BNMFs whereas, the liberated oxygen from B_2_O_2_ is captured by the metalic catalysts and retain back its original form as it was first used in the reactant at the 1^st^ stage.

The as-synthesized microflakes have all the desired characteristics needed for a higher efficiency solid state neutron detectors. In a solid state neutron detector, the active material should have large cross-sections for thermal neutron and can easily produce charged particles due to interaction of neutron. Such a material is then coated on a semiconductor layer so that the charged particles produced by the boron coated material can easily be accelerated into the semiconductor layer to produce electron-hole pair. In such a case the thickness of the boron-coated material must be sufficient enough to capture all incoming neutron flux and at the same time thin enough to allow the daughter nuclei into the semiconductor layer to produce electron-hole pair. The synthesis of layered structures of h-BN with highly crystalline nature were suggested to be the best option in this regard^5^. This demand has been fulfilled by the synthesis of highly crystalline multilayered structures of BNMFs in the present study. The as-synthesized BNMFs has boron-10 (^10^B) with a large cross-section of 3840 b for thermal neutron. This constitutes ~20% of natural boron in h-BN whereas the rest consists of boron-11(^11^B)^22^,^23^. Thus, BNMFs are suggested to be used as a sensing element in a solid state neutron detector. The incoming neutron flux will produce charge particles (daughter nuclei) when interact with BNMFs. Being large band gap semiconductor, BNMFs do not need any other separate semiconductor layer for the production of electron-hole pairs. The as-produced charged particles (α, Li) will then be accelerated in to the same layers of BNMFs and will produce electron-hole pairs^5^. The range of the charge particles corresponds to the thickness of the as-synthesized BNMFs (2–5 μm). Thus their energy will be fully utilized in the production of electron-hole pairs. These pairs will then be detected by their respective electrodes in the detector and will finally be shown in the form of electrical signals on the screen. The BNMFs-based solid state detector thus made is hoped to have the best neutron detection efficiency as compared to any other solid neutron detector.

## Conclusions

Microflakes of h-BN with a thickness or diameter in the range of 2–5 μm and length of greater than 40 μm can be synthesized in the presence of N_2_ as a reaction atmosphere. The precursors, catalysts and gases are chosen in such a way that it can provide a continues, relatively short, simple and efficient way for the synthesis of boron nitride microflakes. It is found that the initial form of the as-used catalysts prepares boron whereas their intermediate state provides nitrogen for the synthesis of boron nitride microflakes. The whole process is extremly safe as no toxic gas or material being utilized during the synthesis. The 2–5 μm thickness and semiconductor nature of the synthesized microflakes makes it a very suitable material as sensing element in a solid state neutron detector with higher detection efficiency.

## Additional Information

**How to cite this article**: Ahmad, P. *et al.* Synthesis of Highly Crystalline Multilayered Boron Niride Microflakes. *Sci. Rep.* **6**, 21403; doi: 10.1038/srep21403 (2016).

## References

[b1] Song L. *et al.* Large scale growth and characterization of atomic hexagonal boron nitride layers. Nano Lett 10, 3209–3215 (2010). 20698639 10.1021/nl1022139

[b2] Wang J. *et al.* Selective synthesis of boron nitride nanotubes by self-propagation high-temperature synthesis and annealing process. Journal of Solid State Chemistry 184, 2478–2484 (2011).

[b3] Pakdel A., Zhi C., Bando Y., Nakayama T. & Golberg D. A comprehensive analysis of the CVD growth of boron nitride nanotubes. Nanotechnology 23, 215601 (2012). 22551670 10.1088/0957-4484/23/21/215601

[b4] Shi Y. *et al.* Synthesis of few-layer hexagonal boron nitride thin film by chemical vapor deposition. Nano Lett 10, 4134–4139 (2010). 20812716 10.1021/nl1023707

[b5] Li J., Dahal R., Majety S., Lin J. & Jiang H. Hexagonal boron nitride epitaxial layers as neutron detector materials. Nuclear Instruments and Methods in Physics Research Section A: Accelerators, Spectrometers, Detectors and Associated Equipment 654, 417–420 (2011).

[b6] Kouzes R. T. *et al.* Neutron detection alternatives to 3He for national security applications. Nuclear Instruments and Methods in Physics Research Section A: Accelerators, Spectrometers, Detectors and Associated Equipment 623, 1035–1045 (2010).

[b7] Pacile D., Meyer J., Girit C. O. & Zettl A. The two-dimensional phase of boron nitride: Few-atomic-layer sheets and suspended membranes. Appl Phys Lett 92, 133107 (2008).

[b8] Han W.-Q., Wu L., Zhu Y., Watanabe K. & Taniguchi T. Structure of chemically derived mono- and few-atomic-layer boron nitride sheets. Appl Phys Lett 93, 223103 (2008).

[b9] Pierson H. O. Boron nitride composites by chemical vapor deposition. Journal of Composite Materials 9, 228–240 (1975).

[b10] Rozenberg A., Sinenko Y. A. & Chukanov N. Regularities of pyrolytic boron nitride coating formation on a graphite matrix. J Mater Sci 28, 5528–5533 (1993).

[b11] Middleman S. The role of gas-phase reactions in boron nitride growth by chemical vapor deposition. Materials Science and Engineering: A 163, 135–140 (1993).

[b12] Zhi C., Bando Y., Tan C. & Golberg D. Effective precursor for high yield synthesis of pure BN nanotubes. Solid State Commun 135, 67–70 (2005).

[b13] Lee C. H., Wang J. S., Kayatsha V. K., Huang J. Y. & Yap Y. K. Effective growth of boron nitride nanotubes by thermal chemical vapor deposition. Nanotechnology 19, 455605 (2008). 21832782 10.1088/0957-4484/19/45/455605

[b14] Pakdel A., Zhi C., Bando Y., Nakayama T. & Golberg D. Boron nitride nanosheet coatings with controllable water repellency. Acs Nano 5, 6507–6515 (2011). 21766852 10.1021/nn201838w

[b15] Lee C. H., Xie M., Kayastha V., Wang J. S. & Yap Y. K. Patterned Growth of Boron Nitride Nanotubes by Catalytic Chemical Vapor Deposition. Chem Mater 22, 1782–1787 (2010).

[b16] Ahmad P., Khandaker M. U. & Amin Y. M. Synthesis of boron nitride nanotubes by Argon supported Thermal Chemical Vapor Deposition. Physica E: Low-dimensional Systems and Nanostructures 67, 33–37 (2015).

[b17] Sinnott S. *et al.* Model of carbon nanotube growth through chemical vapor deposition. Chemical Physics Letters 315, 25–30 (1999).

[b18] Su C.-Y. *et al.* Large-scale synthesis of boron nitride nanotubes with iron-supported catalysts. The Journal of Physical Chemistry C 113, 14732–14738 (2009).

[b19] Zhong B., Song L., Huang X. X., Wen G. W. & Xia L. Synthesis of boron nitride nanotubes with SiC nanowire as template. Mater Res Bull 46, 1521–1523 (2011).

[b20] Arenal R. *et al.* Raman spectroscopy of single-wall boron nitride nanotubes. Nano Lett 6, 1812–1816 (2006). 16895378 10.1021/nl0602544

[b21] Bencic S. Ammonia synthesis promoted by iron catalysts. Literature Report. Dept. of Chemistry, Michigan State University (2001).

[b22] Han W.-Q., Todd P. J. & Strongin M. Formation and growth mechanism of 10 BN nanotubes via a carbon nanotube–substitution reaction. Appl Phys Lett 89, 173103 (2006).

[b23] Reed B. C. the Physics of the Manhattan Project. (Springer, 2010).

